# Knowledge, Attitudes, and Willingness Toward Organ Donation Among High School Students and Teachers in the West Bank, Palestine: A Cross‐Sectional Study

**DOI:** 10.1002/hsr2.71774

**Published:** 2026-01-26

**Authors:** Mahmoud Abu Mayaleh, Ahmad Khleif, Kenana Altell, Abdallah Najjar, Rami Shrouf, Roba Alzuhoor, Abdelrazzaq Abu Mayaleh, Beesan Maraqa, Mohamad Khleif

**Affiliations:** ^1^ Hebron University Hebron Palestine; ^2^ College of Medicine Al‐Quds University Jerusalem Palestine; ^3^ College of Medicine Hebron University Hebron Palestine; ^4^ Faculty of Allied Medical Sciences Palestine Ahliya University Bethlehem Palestine

**Keywords:** knowledge, organ donation, students and teachers

## Abstract

**Background and Aims:**

Organ donation is a vital component of modern healthcare, yet its acceptance varies across populations. Understanding perceptions among future generations and educators is crucial for effective awareness initiatives. This study aimed to assess the knowledge, attitudes, and willingness regarding organ donation among high school students and teachers in the West Bank, Palestine.

**Methods:**

A cross‐sectional study was conducted among 508 participants (370 high school students and 138 teachers) between March and May 2025. Data were collected via a structured online questionnaire following ethical approval. Electronic informed consent was obtained, and anonymity was maintained. Statistical analysis included descriptive statistics, independent‐samples *t*‐tests, and one‐way analysis of variance.

**Results:**

Participants reported moderate mean scores for attitudes (24.16 ± 7.995) and knowledge (6.87 ± 1.598). Students had significantly higher mean knowledge scores than teachers (7.01 vs. 6.51; *p* = 0.004). Willingness to donate to strangers (students: 20.0%, teachers: 12.3%) or register at a donation center (students: 28.4%, teachers: 39.1%) was low. Among students, gender was significantly associated with knowledge (*p* < 0.001) and attitudes (*p *= 0.02). Among teachers, urban residence was associated with knowledge (*p* = 0.002) and attitudes (*p *= 0.03). Key influencing factors included family support, religious guidance, and low trust in the healthcare system.

**Conclusion:**

This study reveals a significant gap between knowledge, attitudes, and willingness to donate organs. Culturally tailored educational programs, religious engagement, and enhanced trust in the healthcare system are essential to promote organ donation in Palestine.

**Abbreviations**
OD: organ donation
WHO: World Health Organization

## 1 Background

Organ donation (OD) is a vital medical and surgical procedure that restores organ function and saves lives. Organs can be obtained from living or deceased donors, most commonly from individuals with confirmed brain death, in accordance with the dead donor rule [1, 2, 3, 4].

Globally, kidney transplants account for about 65% of all procedures, followed by liver (24%), heart (6%), and lung (4%) transplants. This distribution indicates a high global demand for kidney transplants, potentially linked to the prevalence of chronic kidney disease, difficulty of other types of organs, lack of experts, and highlights the relative rarity of transplants for other organ types [5, 6].

Despite advances, the global need for organ transplants far exceeds availability. In 2017, the World Health Organization estimated that only 10% of global demand for solid organ transplants was met [7, 8, 9]. Thus, expanding public awareness and willingness to donate remains a major global challenge [9]. Similar to other regions, Palestine faces limited organ donation, constraining the implementation of lifesaving transplant procedures. Attitudes toward organ donation involve personal beliefs and emotions, while willingness reflects the actual intent to donate. Numerous factors, such as lack of knowledge, gender, education, religious uncertainty, family beliefs, and trust in healthcare systems, affect donation decisions [10, 11].

Evidence shows that increased knowledge fosters positive attitudes and greater readiness to donate [5, 7, 8, 9, 12]. Therefore, educational initiatives are key to improving organ donation rates.

In Arab countries, awareness and willingness to donate remain comparatively low. Surveys in the United States and Europe report willingness rates of 69% and higher, whereas the Middle East averages around 50% [13, 14, 15]. The level of knowledge about both living and deceased organ donation within Arab communities remains unclear. Previous studies have highlighted significant confusion regarding living donations and deceased donations following brain death [16, 17, 18]. In Saudi Arabia, more than 1500 organ transplants are performed annually [19]. While living donors, who are typically biologically related to recipients, account for a substantial portion (75%) of organ donations [20], they continue to play a critical role in the country's transplant efforts. In Palestine, and to the best of our knowledge, no studies have addressed this issue thus far.

Organ donation in Palestine is shaped by a complex mix of ethical, legal, socioeconomic, and political factors. Challenges like poverty, cultural beliefs, travel restrictions, and legislative gaps hinder the development of a robust donation framework [21, 22].

Cultural and religious misconceptions often impede willingness to donate. However, a key 2013 fatwa permits organ transfers from both living and deceased donors under strict ethical guidelines, helping to align religious beliefs with medical needs [18, 23, 24, 25].

Palestine's legal framework for transplantation was underdeveloped until recently. Laws passed in 2017 and 2018 introduced regulations, banned organ trafficking, and improved medical protection. Despite this progress, cultural resistance and incomplete bylaws mean significant challenges persist in implementing these procedures [26, 27, 28, 29, 30, 31, 32, 33].

Adolescents are an important target group for organ donation education because adolescence is a key stage in the development of human values, and a positive attitude toward organ donation instilled in adolescence will last a lifetime. In addition, adolescent education can promote discussions with family members about organ donation [34, 35]. Various studies have discussed the strong correlation between attitudes and willingness, which suggests that increased public awareness and education on organ transplantation could further increase donation rates. A study conducted in Iran demonstrated that the attitudes of 416 Iranian high‐school girls toward organ donation were highly positive; 92% of participants were willing to donate organs after death, whereas 47% were willing to donate while alive [36].

Moreover, a study conducted in Turkey explored the impact of education on high school students' attitudes toward organ donation. Initially, only 24.9% of the students expressed a willingness to donate organs after death, and 14.3% of them were absolutely opposed to donation. However, after an educational program, the percentages changed to 38.4% and 7.2%, respectively [37].

However, Teachers also play a critical role in shaping students ideas toward organ donation. A study conducted in Turkey examined the knowledge, attitudes, and behaviors of schoolteachers regarding organ donation. While an impressive 96.6% considered organ donation to be a compassionate act, only 0.7% had actually donated an organ. Furthermore, 66.5% did not consider future donations. These results reveal a significant disconnect between awareness and action, underscoring the need for improved education and motivation to foster informed choices about organ donation [38].

Despite global efforts, there remains a gap in understanding Palestinian perspectives on organ donation. Given the region's cultural and religious context, this study aims to assess the knowledge, attitudes, and willingness of high school students and teachers in the West Bank toward organ donation, providing evidence to inform culturally sensitive awareness and policy initiatives.

## 2 Methods

### 2.1 Study Design, Time, and Setting

This research employs a descriptive cross‐sectional study design to assess the knowledge, attitudes, and willingness of high school students and teachers in Palestine toward organ donation. Data collection took place from March 2025 to May 2025, and an online questionnaire was used to efficiently reach a larger and more diverse population within the West Bank.

### 2.2 Sampling Technique and Sample Size

The study population included Palestinian high school students in grades 11 and 12 (Tawjihi) aged from 15 to 18, and teachers across Palestine. Participants were recruited using a convenience sampling strategy through an online questionnaire distributed via official Ministry of Education channels. The sample size for this study was determined on the basis of data from the Palestinian Ministry of Education. According to the most recent statistics, the total number of high school (Tawjihi) students is approximately 50,000, and 11th‐grade students number approximately 58,000 [39]. The total number of teachers is 77,234, distributed as follows: 54,081 in government schools, 12,183 in UNRWA schools, and 10,970 in private schools [40]. The sample size for the student population was estimated using the single‐proportion formula:

$$n=\frac{Z^{2}p(1-p)}{E^{2}},$$

where *Z *= 1.96*Z* corresponds to a 95% confidence level, *p* = 0.5 represents an assumed prevalence (used to maximize sample size in the absence of prior data), and *E* = 0.05 denotes the acceptable margin of error. This yielded an initial sample size of 384 students. Because the student population is finite (approximately 50,000 students), the finite population correction (FPC) was applied, resulting in a corrected minimum sample of approximately 381 students.

A total of 436 students were initially recruited. Among these, 61 students (14.0%) declined to provide informed consent. Further exclusions were applied to ensure that only participants without direct personal experiences that might bias their perceptions of organ donation were included. Specifically, 3 students (0.7%) were excluded for being patients in need of an organ transplant, 2 (0.5%) for having previously donated an organ, and 3 (0.7%) for having undergone an organ transplant procedure. After all exclusions, the final analytical sample comprised 370 student participants. Although this number falls slightly below the lower bound of the target range, it provides a reasonably precise estimate for the outcomes of interest within this population.

Given the substantial difference in total population and professional characteristics, the teacher sample size was calculated independently using the same single‐proportion formula. With *Z* = 1.96, *p* = 0.5, and *E* = 0.05, the initial calculated sample was 384 teachers. Applying the finite population correction for the total teacher population (*N* = 77,234) yielded an adjusted minimum of approximately 382 teachers.

In practice, recruitment of teachers proved substantially more challenging than anticipated. Despite coordination with multiple educational directorates, only 166 teachers could be approached. Among them, 23 (13.9%) declined to provide informed consent. In addition, 5 teachers (3.0%) were excluded for being patients awaiting organ transplantation, 1 (0.6%) for having previously donated an organ, and 2 (1.2%) for having undergone an organ transplant procedure. Consequently, the final analytical sample included 138 teachers.

The shortfall from the planned sample size is primarily attributed to logistical and contextual challenges specific to the Palestinian setting. These include restricted access to certain geographic areas, security checkpoints, fragmented school jurisdictions (governmental, UNRWA, and private), and limited administrative cooperation due to the political situation. Furthermore, time constraints during the academic year and overlapping examination schedules reduced teacher availability for participation. As a result, the teacher sample should be interpreted as exploratory, and findings from this subgroup may have wider confidence intervals and lower statistical precision compared to those from the student population.

### 2.3 Instrument and Pilot Testing

The questionnaire used in this study was composed of four sections: the Organ Donation Knowledge Questionnaire (ODKQ), the Attitudes Toward Organ Donation Scale (ATODS), the Willingness to Donate Organs Questionnaire (WDOQ), and a demographic information form (DIF). All the components were originally derived from previously validated instruments reported in the literature [9, 41, 42].

However, the questionnaire was adapted to suit the cultural and social context of the Palestinian community. The adapted version was reviewed by two field experts to ensure content validity and cultural appropriateness, Also, these tools were adapted and translated to Arabic to be understandable by Palestinian students and teachers. A pilot study was conducted with a sample of 30 participants (students and teachers) to assess the clarity, relevance, and applicability of the questionnaire items. On the basis of the feedback received, necessary modifications were made to enhance question clarity and contextual relevance. The 650 responses initially collected included data from these pilot study participants, which were subsequently excluded from the final analytical sample to ensure data integrity. The revised questionnaire was then administered in the main study.

Reliability of the tool was measured by calculating the Cronbach Alpha in the study. Alpha among the total sample size was 0.81, which indicates a high level of reliability of the tool. The total attitude scale scores ranged from 9 to 45 (mean = 25.1), and the knowledge scale scores ranged from 1 to 10 (mean = 6.9), while the willingness total score ranged from 0 to 8 (mean = 2.7). High scores indicate better attitude, knowledge and willingness towards organ donation.

### 2.4 Data Collection Procedures

In this study, a crucial collaboration was established with the Ministry of Education and Higher Education in Palestine. This partnership facilitated the distribution of the online questionnaire to ensure comprehensive coverage across various geographic regions and school districts. The questionnaire link was disseminated through official channels managed by the Ministry, which reached designated schools and teachers. A consent form was integrated into the questionnaire to inform participants about the study's objectives, emphasizing that the collected data would be used solely for research purposes. Additionally, strict measures were implemented to maintain the confidentiality and anonymity of participants' responses throughout the research process.

### 2.5 Measurement Tool Description

Data were collected using a structured, self‐administered questionnaire that was adapted from previously validated instruments and reviewed by a panel of local experts to ensure cultural and linguistic appropriateness.

The questionnaire comprised four sections. The first section gathered demographic information, including age, gender, religion, governorate, residence type, and educational level, and contained health‐related exclusion criteria. The second section measured attitudes toward organ donation using a nine‐item, five‐point Likert scale (1 = strongly disagree to 5 = strongly agree). The third section assessed participants' willingness to donate across eight different contexts using a dichotomous (yes/no) response format. The final section evaluated knowledge of organ donation via ten true/false statements, with scores calculated from the total number of correct answers.

Validity of the tool was measured through expert review.

### 2.6 Statistical Analysis

All the collected data were subjected to thorough screening and cleaning prior to analysis. This process included identifying and managing missing values and rectifying illogical entries, such as an improbable age recorded for a student participant. Data management involved several transformation and recoding steps. Specifically, the variable “Governorate” was recoded into “Geographical Region” (North; South/Central) to facilitate broader regional analysis. Similarly, “Residency” was recoded into “Type of Residence (Urban/Rural)” (Urban; Rural) to categorize participants' living environments more effectively. Furthermore, “Age_Numeric” was categorized into “Age Category” (< 30 years; 30–39 years; > = 40 years) for specific analyses.

Descriptive statistics were used to summarize the demographic characteristics of the participants and the overall distributions of knowledge, attitudes, and willingness toward organ donation. Frequencies and percentages were computed for categorical variables (gender, geographical region, type of residence, education level, and age category). The mean (*M*), standard deviation (SD), minimum, and maximum values were calculated for continuous variables (Age_Numeric, total attitude score, and total knowledge score).

To assess the associations between demographic characteristics and total attitude/knowledge scores, inferential statistical tests were employed. Independent‐samples *t*‐tests were conducted to compare the mean total attitudes and knowledge scores between two‐category demographic variables (gender and type of residence). For demographic variables with more than two categories (age category, geographical region, and education level), one‐way analysis of variance (ANOVA) and a *t*‐test were performed to compare the mean total attitudes and knowledge scores across groups. Post hoc Tukey's HSD tests were used where ANOVA revealed a statistically significant difference to identify specific group differences. All statistical tests were two‐sided, and the a priori level of statistical significance was set at *p* < 0.05. All analyses were pre‐specified. The statistical analyses were performed using IBM SPSS Statistics (Version 23).

### 2.7 Ethics Approval and Consent to Participate

The study strictly adhered to ethical standards for research involving human participants, as established by Hebron University. Approval from the Institutional Review Board (IRB) of the College of Medicine at Hebron University was secured on March 03, 2025, with the approval reference number ER. CM.32.2025.

The participants were thoroughly informed of the study's objectives, procedures, and the voluntary nature of their participation, with the assurance that they might withdraw at any point without penalty. Informed consent was obtained electronically from all adult participants. For high school students under the age of 18, permission to participate was secured from their respective school administrations, with the students themselves providing their assent and voluntary participation.

To protect participant anonymity and privacy, no personal identifiers were collected. All the data obtained were exclusively used for research purposes and handled with utmost confidentiality. The data were stored on a secure, encrypted cloud platform and will be retained for a period of 5 years as per institutional guidelines. Data access was limited to authorized members of the research team, ensuring secure and confidential handling of information throughout the study.

## 3 Results

### 3.1 Demographic Characteristics of the Participants

The final analytical sample for the study comprised a total of 508 participants. This sample included 370 student participants, representing 72.8% of the total, and 138 teacher participants, constituting 27.2%. The detailed demographic characteristics of both groups are presented in Table 1.

**Table 1:** Demographic characteristics of the participants (*n* = 508).

Characteristic	Category	Students *n*, (%)	Teachers *n*, (%)
Age	Mean ± SD	16.95 ± 690	41.30 ± 8.389
	Minimum–maximum	15–18	24–59
Gender	Female	291, (78.6)	88, (63.8)
	Male	79, (21.4)	50, (36.2)
	Total	370, (100.0)	138, (100.0)
Geographical region	North	249, (67.3)	86, (62.3)
	South/Central	121, (32.7)	39, (28.3)
	Total valid	370, (100.0)	125, (90.6)
	(Missing)		13, (9.4)
Type of residence (urban/rural)	Urban	106, (28.6)	36, (26.1)
	Rural	264, (71.4)	102, (73.9)
	Total	370, (100.0)	138, (100.0)
Highest educational level achieved?	High school	370, (100.0)	N/A
	Associate diploma (2 years posthigh school)	N/A	8, (5.8)
	University degree (Bachelor's)	N/A	92, (66.7)
	Graduate studies (Master's/PhD)	N/A	38, (27.5)
	Total	370, (100.0)	138, (100.0)

*Note:* This table presents the frequency and percentage distributions of demographic characteristics for both the student and teacher participants in the study.

The student cohort (*N* = 370) had a mean age of 16.95 years (SD = 0.690), with ages ranging from 15 to 18 years. Females constituted the majority within this group, accounting for 78.6% (*n* = 291), whereas males constituted 21.4% (*n* = 79). Geographically, most students (67.3%, *n* = 249) were from the North region, with 32.7% (*n* = 121) residing in the South/Central regions. In terms of residential setting, 28.6% (*n* = 106) of the students lived in urban areas, and a larger proportion, 71.4% (*n* = 264), resided in rural areas. All the student participants were enrolled at the high school level (100.0%, *n* = 370).

Among the teacher participants (*N* = 138), the mean age was 41.30 years (SD = 8.389), with ages ranging from 24 to 59 years. Compared with males, females constituted the majority in this group (63.8%, *n* = 88) (36.2%, *n* = 50). Geographically, 62.3% (*n* = 86) of the teachers were from the North region, and 28.3% (*n* = 39) were from the South/Central regions; however, data for 13 teachers (9.4%) were missing for this variable. In terms of residential setting, 26.1% (*n* = 36) lived in urban areas, whereas 73.9% (*n* = 102) resided in rural areas. The educational background of the teachers indicated that 66.7% (*n* = 92) held a university degree (Bachelor's degree), 27.5% (*n* = 38) had pursued Graduate studies (Master's/PhD), and 5.8% (*n* = 8) held an associate diploma (2 years posthigh school).

### 3.2 Willingness to Donate Organs

The students' willingness to engage in organ donation is summarized in Table 2. Approximately 41.9% of the students indicated that they would take organ donation more seriously if they were contacted by a trusted organization. However, only 28.4% stated that they would register if an organ donation center existed in Palestine. When considering religious guidance, 47.3% reported that they would be willing to consider donation after discussing the matter with a religious scholar. Familial support was reported to be significant, with 58.9% of the students stating that family support would influence their decision to donate. Willingness to travel abroad for organ donation or transplantation was low, with only 15.1% affirming this. Nearly half (48.9%) stated that they would be more willing to donate if they were allowed to choose simpler organs such as the skin. With respect to altruistic donation to strangers, only 20.0% reported having considered donating an organ to someone they do not know. Finally, 27.8% expressed trust in the Palestinian healthcare system concerning organ donation or transplantation procedures.

**Table 2:** Willingness to donate organs (*n* = 508).

Statement	Student		Teacher	
	Yes (%)	No (%)	Yes (%)	No (%)
Would you take organ donation more seriously if you were contacted by a trusted organization?	41.9	58.1	46.4	53.6
If there was an organ donation center in Palestine, would you register?	28.4	71.6	39.1	60.9
Would you consider organ donation after discussing the matter with a religious scholar?	47.3	52.7	51.4	48.6
Does family support for your desire to donate affect your decision?	58.9	41.1	54.3	45.7
Would you consider traveling abroad for organ donation or transplantation?	15.1	84.9	7.2	92.8
Would you be more willing to donate if you could choose to donate simple organs like skin only?	48.9	51.1	59.4	40.6
Have you ever considered donating one of your organs to a stranger you do not know?	20	80	12.3	87.7
Do you trust the Palestinian healthcare system concerning organ donation or transplantation procedures?	27.8	72.2	26.8	73.2

*Note:* This table presents the percentage of participants indicating “yes” or “no” for each willingness statement among both the student and teacher groups.

Among the teacher participants, their willingness to engage in organ donation is also detailed in Table 2. A total of 46.4% of the teachers stated that they would consider organ donation more seriously if they were approached by a trusted organization. For the presence of an organ donation center in Palestine, 39.1% indicated that they would register. When considering religious guidance, 51.4% reported that they would be willing to consider donation after discussing the matter with a religious scholar. Familial support was noted as influencing decisions for 54.3% of the teachers. Willingness to travel abroad for organ donation or transplantation was low, with only 7.2% affirming this. A larger percentage (59.4%) were willing to donate skin if they could choose simpler organs. The consideration of donating an organ to a stranger was low, with only 12.3% reporting that they had considered it. Finally, 26.8% expressed trust in the Palestinian healthcare system concerning organ donation or transplantation procedures.

### 3.3 Overall Attitude and Knowledge Scores

Table 3 presents the overall descriptive statistics for the total attitude and knowledge scores across all study participants, disaggregated by participant type, including an overall summary for the combined sample.

**Table 3:** Overall attitude and knowledge scores (*n* = 508).

Score type	Statistic	Overall (*N* = 508)	Students (*n* = 370)	Teachers (*n* = 138)	*p* value
Attitude	Mean (SD)	24.16 (7.995)	24.43 (8.107)	23.45 (7.666)	0.22
	Min	9	9	9	
	Max	45	45	45	
Knowledge	Mean (SD)	6.87 (1.598)	7.01 (1.497)	6.51 (1.797)	0.004
	Min	1	1	2	
	Max	10	10	10	

*Note:* This table presents the descriptive statistics for the total attitude and knowledge scores among the student and teacher participants.

For the total attitude score, the student participants (*N* = 370) presented a mean score of 24.43 (SD = 8.107), with scores ranging from 9 to 45. The teacher participants (*N* = 138) had a mean attitude score of 23.45 (SD = 7.666), with scores ranging from 9 to 45. Overall, for the entire sample (*N* = 508), the mean total attitude score was 24.16 (SD = 7.995), with scores ranging from 9 to 45. The *p* value for the comparison between the student's and teacher's mean attitude scores was 0.22.

With respect to the total knowledge score, the student participants (*N* = 370) achieved a mean score of 7.01 (SD = 1.497), with scores ranging from 1 to 10. The teacher participants (*N* = 138) had a mean knowledge score of 6.51 (SD = 1.797), with scores ranging from 2 to 10. For the overall sample (*N* = 508), the mean total knowledge score was 6.87 (SD = 1.598), with scores ranging from 1 to 10. The *p* value for the comparison between the student and teacher mean knowledge scores was 0.004.

### 3.4 Associations of Demographic Characteristics With Total Attitudes and Knowledge Scores

Table 4 presents the associations between various demographic characteristics and the total attitudes and knowledge scores of the student and teacher participants.

**Table 4:** Mean attitudes and knowledge scores by demographic characteristics of student and teacher participants, with associated *p* values.

Characteristic	Category	Attitude				Knowledge			
		Students		Teachers		Students		Teachers	
		Mean (SD)	*p* value	Mean (SD)	*p* value	Mean (SD)	*p* value	Mean (SD)	*p* value
Age	< 30 years	N/A	N/A	22.00 (7.913)	0.741	N/A	N/A	6.14 (1.610)	0.711
	30–39 years			23.53 (8.118)				6.60 (1.932)	
	≥ 40 years			23.75 (7.602)				6.51 (1.750)	
Gender	Male	22.53 (5.963)	0.02	24.16 (7.206)	0.402	6.47 (1.980)	< 0.001	6.46 (2.012)	0.789
	Female	24.95 (8.545)		23.05 (7.928)		7.15 (1.303)		6.55 (1.674)	
Geographical region	North	24.96 (8.575)	0.07	23.43 (8.177)	0.943	6.93 (1.545)	0.159	6.31 (1.874)	0.224
	South/Central*	23.35 (6.958)		23.54 (7.052)		7.17 (1.387)		6.74 (1.697)	
Type of residence (Urban/rural)	Urban	24.08 (7.545)	0.60	25.81 (7.604)	0.03	6.81 (1.768)	0.15	7.22 (1.456)	0.002
	Rural	24.57 (8.332)		22.62 (7.551)		7.08 (1.369)		6.26 (1.845)	
Highest educational level achieved?	High school	N/A	N/A	N/A	N/A	N/A	N/A	N/A	N/A
	Associate diploma (2 years posthigh school)			20.75 (5.148)	0.588			4.75 (1.035)	0.004
	University degree (Bachelor's)			23.55 (7.725)				6.47 (1.794)	
	Graduate studies (Master's/PhD)			23.76 (8.008)				7.00 (1.708)	

For students, a statistically significant difference in attitude was observed based on gender (*p* = 0.02), with female students (mean = 24.95, SD = 8.545) exhibiting a slightly higher mean attitude score than male students (mean = 22.53, SD = 5.963). While not statistically significant, there was a trend for geographical region (*p* = 0.07), with students from North Palestine (mean = 24.96, SD = 8.575) having a somewhat higher mean attitude score than those from South China/Central Palestine (mean = 23.35, SD = 6.958). No significant differences were found in student attitudes based on age, type of residence, or highest educational level achieved.

Conversely, among teachers, a significant difference in attitudes was noted on the basis of their type of residence (*p* = 0.03). Teachers residing in urban areas (mean = 25.81, SD = 7.604) reported a higher mean attitude score than did those in rural areas (mean = 22.62, SD = 7.551). No statistically significant differences were observed in teacher attitudes concerning age, gender, geographical region, or highest educational level achieved.

With respect to the knowledge scores of the students, a statistically significant difference in knowledge was observed across genders (*p* < 0.001). Compared with male students, female students (mean = 7.15, SD = 1.303) presented a significantly higher mean knowledge score (mean = 6.47, SD = 1.980). No significant differences were found in student knowledge levels based on age, geographical region, type of residence, or highest educational level achieved.

With respect to teachers, statistically significant associations with knowledge level were found for both type of residence (*p* = 0.002) and highest educational level achieved (*p* = 0.004). Teachers residing in urban areas (mean = 7.22, SD = 1.456) had significantly higher knowledge scores than did those in rural areas (mean = 6.26, SD = 1.845). In terms of educational level, a statistically significant association was observed (*p* = 0.004). Tukey's post hoc test revealed more knowledge among teachers with bachelor's or postgraduate degrees than among those with diploma degrees (*M* = 6.5, *p* = 0.02; *M* = 7, *p* = 0.003, respectively). No significant differences were observed in teacher knowledge concerning age, gender, or geographical region.

Regression models analysis for the sample data revealed no new findings apart from those of the bivariate analysis, except for the knowledge and attitude levels for the teachers, where the regression models revealed no significant effect of residency on teachers knowledge or attitude (beta = −0.13, *p* = 0.10; beta = −0.06, *p* = 0.44, respectively).

## 4 Discussion

This study provides the first comprehensive assessment of knowledge, attitudes, and willingness toward organ donation among high school students and teachers on the West Bank, Palestine. The findings offer crucial insights into the local context, highlighting both alignment with global trends and unique regional patterns influencing organ donation decisions. Given the persistent global organ shortage, understanding the societal factors that facilitate or impede donation, particularly in under researched regions such as Palestine, is vital.

The mean knowledge scores in this study were moderate (7.01 for students and 6.51 for teachers out of 10). This aligns with findings from other Middle Eastern countries, where a meta‐analysis reported pooled overall knowledge regarding organ donation at 69% [15]. Similar moderate to high awareness rates (70%–90%) have been reported in Saudi Arabia [43], China [44], and Ethiopia [45], whereas some Latin American studies reported lower knowledge (28.6%) [46]. The relatively greater knowledge among students than among teachers may indicate a generational shift in information access and receptiveness, suggesting that digital platforms could be effective for youth campaigns, whereas older demographics might require different approaches.

No statistically significant difference in attitude scores was found between students (mean: 24.43) and teachers (mean: 23.45) (*p* = 0.22). This suggests a similar overall sentiment toward organ donation across both groups, despite knowledge disparities. Positive attitudes are widely reported globally (e.g., 91.9% in Nicaragua [46] and 82% in Germany [47]), often at higher rates than actual willingness. The consistent attitude scores, despite knowledge differences, underscore the complexity of attitude formation, which is deeply rooted in cultural, religious, and personal values. This implies that educational interventions need to engage with emotional, ethical, and spiritual dimensions, moving beyond simple information dissemination.

Willingness varied by scenario: notably low for donating to strangers (students: 20.0%, teachers: 12.3%) and for registering if a center existed in Palestine (students: 28.4%, teachers: 39.1%). However, willingness was greater for simpler organs such as the skin (students: 48.9%, teachers: 59.4%). Global willingness rates vary significantly (e.g., 49.8% in the Middle East [15] and 76% in Spain [48]). The gap between knowledge/attitudes and actual willingness in Palestine, particularly for whole organs or strangers, represents a critical barrier. This finding indicates that practical steps toward commitment are hindered by specific, deeply rooted concerns about body integrity, healthcare system trust, and the nature of altruism. Strategies must address these specific fears and promote a broader understanding of altruism, possibly via a phased approach focusing on simpler donations first.

Gender emerged as a significant factor among the students, with female students (mean = 7.15, SD = 1.303) demonstrating significantly greater knowledge (*p* < 0.001) and more positive attitudes (mean = 24.95, SD = 8.545, *p* = 0.02) than male students did (knowledge mean = 6.47, SD = 1.980; attitude mean = 22.53, SD = 5.963). This gender difference was not observed among teachers.

This finding for female students aligns with international trends where women often express greater willingness and more favorable attitudes toward organ donation and are more likely to discuss it for altruistic reasons. Data from the US also indicate a higher proportion of female living donors (55.6%–58%) [49]. A recent study also highlighted that women were more willing to donate organs to family members and strangers than men were [50].

For students, residence type was not statistically significant for knowledge or attitudes (*p* > 0.05). This contrasts with some international studies where urban residents presented greater knowledge and attitudes [46]. This novel pattern in Palestine may reflect pervasive digital media and uniform educational curricula for this younger demographic, suggesting that broad‐based digital campaigns and consistent school content can be effective. Among teachers, urban residence was statistically significant for both knowledge (*p* = 0.002) and attitudes (*p* = 0.03), with urban teachers being more informed and positive. This aligns with international findings [46], highlighting persistent disparities for adult professionals in residential settings. Targeted interventions for teachers should prioritize rural areas, providing educational resources to them.

Compared with teachers, students had significantly higher knowledge scores (*p* = 0.004) but no significant difference in attitude scores (*p* = 0.22). This suggests that knowledge alone is insufficient to shape a positive attitude. This pattern indicates a generational gap: younger individuals (students) may be more receptive to new information, whereas attitudes in older individuals (teachers) are more entrenched. Educational strategies should be age‐group specific: for students, they should focus on translating knowledge into positive attitudes by addressing underlying fears; for teachers, they should tailor interventions to their specific concerns and leverage their influential community role.

The observed data reveal that the demographic factors influencing attitudes and knowledge are not uniform across students and teachers. Gender significantly affects both the attitudes and knowledge of students, but this influence is not present for teachers. Conversely, the type of residence significantly impacts both attitudes and knowledge for teachers but not for students. Additionally, educational level is a significant factor for teacher knowledge but not for student knowledge. This differential impact suggests that life experiences, social roles, and exposure to information unique to each group (students *vs.* teachers) modulate the influence of these demographic variables. For example, teachers' professional environment, which might vary significantly between urban and rural settings, could provide differential access to health information or discussions, explaining the residence effect. For students, gender differences might reflect early socialization patterns or varying levels of engagement with health‐related topics. This differential impact necessitates tailored interventions rather than a one‐size‐fits‐all approach.

The finding that students, despite having greater knowledge, do not exhibit significantly more positive attitudes reinforces that knowledge alone is insufficient to shape attitudes. This “knowledge–attitude gap” is globally documented [51], as seen in contexts such as India, where positive attitudes do not translate into high willingness [52], and Ethiopia, where high knowledge among medical students coexists with low positive attitudes and willingness [45]. The Palestinian context suggests that factual information is insufficient to overcome deeply ingrained sociocultural, religious, and psychological barriers. Attitudes are shaped by familial beliefs, religious interpretations, and societal norms that prioritize body integrity. This gap highlights that an attitude shift requires the transformation of deeply held values, often through community dialog and endorsement from trusted figures. Public health campaigns must evolve from purely informational approaches to culturally sensitive interventions addressing emotional, ethical, and religious concerns, fostering collective responsibility and trust.

The study demonstrated the profound influence of family support (58.9% of students, 54.3% of teachers) and consulting religious scholars (47.3% of students, 51.4% of teachers) on organ donation decisions on the West Bank. This aligns with research across the Middle East and Asia, where family and religious beliefs are paramount determinants of organ donation willingness [15]. A recent study indicates decisions about deceased organ donation in Muslim communities tend to be collective, shaped by religious views and uncertainty rather than purely individual choices [53]. This underscores that organ donation in Palestine is a deeply embedded socioreligious decision. Top‐down campaigns will be ineffective without grassroots engagement with religious institutions and community elders. Gaining their endorsement and equipping them as advocates is crucial, as decisions are often collective and require communal validation.

A striking finding is the exceptionally low level of trust in the Palestinian healthcare system concerning organ donation (27.8% of students, 26.8% of teachers). This formidable barrier is consistent with international literature emphasizing public trust as “utterly indispensable” [54], with greater public distrust being directly associated with reduced donation rates [55]. The low level of trust in Palestine may stem from perceived inefficiencies, a lack of transparency, or broader sociopolitical instability. This suggests that, rather than merely focusing on public education, any successful organ donation program must prioritize systemic reforms to increase transparency, accountability, and perceived quality of care. Without addressing this foundational issue, other efforts will likely yield limited results.

The study revealed a significantly greater willingness to donate “simple organs” (48.9% of the students, 59.4% of the teachers) than to donate very little to strangers (20.0% of the students, 12.3% of the teachers). This pattern is consistent with international research where concerns about body disfigurement or emotional attachment influence willingness [56]. The reluctance to donate to strangers is also globally prevalent [57], suggesting that altruism in collectivist societies is often bounded by social familiarity. Strategies must address specific fears about bodily desecration and, for nondirected donation, articulate a compelling vision of universal altruism that resonates locally, perhaps by framing it as a broader act of compassion.

The findings are deeply intertwined with the unique cultural, religious, and sociopolitical landscape of the West Bank. Palestine is a predominantly Muslim society where religious interpretations, particularly regarding the sanctity of the human body and the afterlife, profoundly influence health decisions. The strong emphasis on family support and consulting by religious scholars aligns with Palestinian collectivism. Low levels of trust in the Palestinian healthcare system (< 28%) likely stem from broader infrastructure challenges and historical experiences. The desire to donate only “simple organs” and the reluctance to donate strangers further manifest cultural and religious sensitivities [56]. The observed differences between students and teachers, particularly the urban–rural divide among teachers, also reflect socioeconomic realities and information access disparities. The study collectively highlights that organ donation is not a universal concept but is deeply shaped by the local sociocultural ecosystem, necessitating a highly localized and culturally sensitive approach. Overall, while Palestinian participants share similar cultural and religious barriers to donation as those reported in other Muslim‐majority regions, unique contextual factors such as political instability and limited healthcare infrastructure further shape public perceptions and trust, distinguishing the Palestinian experience from other international contexts.

## 5 Strengths and Limitations

### 5.1 Strengths of the Study

This study is the first of its kind in Palestine to include both high school students and teachers, providing novel perspectives and a unique comparative approach to generational and professional influences. It utilized a large and well‐distributed sample of 508 participants from multiple regions within the West Bank, enhancing representativeness and contributing to the statistical power and reliability of the results, providing a strong baseline for future research.

## 6 Limitations of the Study

It is important to acknowledge several limitations of this study. The cross‐sectional design can only show associations between variables and cannot establish causality, underscoring the need for future longitudinal studies to track changes over time. The generalizability of the findings is also limited, as the sample was confined to the educational community students and teachers and does not reflect the broader Palestinian population. Recruitment challenges resulted in a smaller teacher sample than planned, reducing the statistical precision for that group. Finally, the study's reliance on self‐administered online questionnaires introduces potential for bias; this includes selection bias, which may underrepresent individuals with poor digital literacy, and response or recall bias, where participants might provide socially desirable or inaccurate information.

## 7 Conclusion

This pioneering cross‐sectional study in the West Bank reveals moderate knowledge and attitudes toward organ donation among high school students and teachers, alongside a significant gap between knowledge and willingness, especially for complex organs and nondirected donation. Key determinants include the profound influence of family and religious guidance and, critically, a pervasive lack of trust in the Palestinian healthcare system. These findings underscore that organ donation is not merely a medical issue but also a complex sociocultural phenomenon deeply embedded in the Palestinian context, requiring tailored interventions.

## 8 Recommendations for Future Research

On the basis of the findings and limitations, several avenues for future research are recommended: longitudinal studies to track changes and assess long‐term impact; qualitative research to explore nuanced beliefs and emotional considerations; interventional studies to evaluate culturally tailored interventions (e.g., involving religious leaders, family‐centric programs, and trust‐building initiatives); and comparative studies to include other Palestinian territories or diaspora populations for broader understanding.

## 9 Practical Recommendations for Public Health Interventions and Policy Development

To address identified barriers and promote organ donation in the West Bank, the following practical recommendations are proposed: culturally sensitive education that engages religious leaders and community elders; family‐centric approaches recognizing their central role; building healthcare Trust through strategic initiatives to enhance transparency and quality; targeted outreach for rural teachers; phased donation promotion starting with “simple organs” to build comfort; and the establishment of a National Organ Donation Program advocating for a well‐regulated, transparent center in Palestine.

## Author Contributions

**Mahmoud Abu Mayaleh:** data collection, analysis, manuscript writing, and review. **Mohamad Khleif:** conception, analysis, and critical manuscript revision. **Ahmad Khleif:** data collection, design of the work, manuscript writing, analysis, and critical manuscript revision. **Abdelrazzaq Abu Mayaleh:** data collection and critical manuscript revision. **Abdullah Najjar:** data collection, interpretation of data, manuscript writing, and critical manuscript revision. **Kenana Altell:** data collection, acquisition, manuscript writing, and critical manuscript revision. **Rami Shrouf:** data collection, manuscript writing, and critical manuscript revision. **Roba Zhoor:** data collection and manuscript writing. **Beesan Maraqa:** conception and critical manuscript revision.

## Funding

The authors received no specific funding for this work.

## Conflicts of Interest

The authors declare no conflicts of interest.

## Transparency Statement

The corresponding author, Kenana Altell, affirms that this manuscript is an honest, accurate, and transparent account of the study being reported; that no important aspects of the study have been omitted; and that any discrepancies from the study as planned (and, if relevant, registered) have been explained.

## Data Availability

The data sets used and/or analyzed during the current study are available from the corresponding author upon reasonable request.
